# Design and Evaluation of a Computer-Based 24-Hour Physical Activity Recall (cpar24) Instrument

**DOI:** 10.2196/jmir.7620

**Published:** 2017-05-30

**Authors:** Simone Kohler, Gundula Behrens, Matthias Olden, Sebastian E Baumeister, Alexander Horsch, Beate Fischer, Michael F Leitzmann

**Affiliations:** ^1^ Department of Epidemiology and Preventive Medicine University of Regensburg Regensburg Germany; ^2^ Institute for Community Medicine University Medicine Greifswald Greifswald Germany; ^3^ Department of Computer Science UiT – The Arctic university of Norway Breivika Tromsø Norway

**Keywords:** web-based method, validity, reliability, usability, lifestyle behavior, physical activity, sedentary behavior

## Abstract

**Background:**

Widespread access to the Internet and an increasing number of Internet users offers the opportunity of using Web-based recalls to collect detailed physical activity data in epidemiologic studies.

**Objective:**

The aim of this investigation was to evaluate the validity and reliability of a computer-based 24-hour physical activity recall (cpar24) instrument with respect to the recalled 24-h period.

**Methods:**

A random sample of 67 German residents aged 22 to 70 years was instructed to wear an ActiGraph GT3X+ accelerometer for 3 days. Accelerometer counts per min were used to classify activities as sedentary (<100 counts per min), light (100-1951 counts per min), and moderate to vigorous (≥1952 counts per min). On day 3, participants were also requested to specify the type, intensity, timing, and context of all activities performed during day 2 using the cpar24. Using metabolic equivalent of task (MET), the cpar24 activities were classified as sedentary (<1.5 MET), light (1.5-2.9 MET), and moderate to vigorous (≥3.0 MET). The cpar24 was administered twice at a 3-h interval. The Spearman correlation coefficient (r) was used as primary measure of concurrent validity and test-retest reliability.

**Results:**

As compared with accelerometry, the cpar24 underestimated light activity by −123 min (median difference, *P* difference <.001) and overestimated moderate to vigorous activity by 89 min (*P* difference <.001). By comparison, time spent sedentary assessed by the 2 methods was similar (median difference=+7 min, *P* difference=.39). There was modest agreement between the cpar24 and accelerometry regarding sedentary (*r*=.54), light (*r*=.46), and moderate to vigorous (*r*=.50) activities. Reliability analyses revealed modest to high intraclass correlation coefficients for sedentary (*r*=.75), light (*r*=.65), and moderate to vigorous (*r*=.92) activities and no statistically significant differences between replicate cpar24 measurements (median difference for sedentary activities=+10 min, for light activities=−5 min, for moderate to vigorous activities=0 min, all *P* difference ≥.60).

**Conclusion:**

These data show that the cpar24 is a valid and reproducible Web-based measure of physical activity in adults.

## Introduction

Physical activity is associated with decreased risk of numerous chronic diseases, including type 2 diabetes [1], cardiovascular disease [2], and certain types of cancer [3]. However, information regarding which frequencies, intensities, and durations of specific activities or combinations of activities are relevant to reducing disease risk is sparse. Thus, comprehensive assessments of physical activity are required to better characterize the relation of physical activity to risk of chronic disease.

A variety of methods to assess physical activity in epidemiologic studies exist, and each measurement technique has particular advantages and limitations [4-6]. Increased availability of the Internet with rising numbers of Internet users and recent progress in computer technology provide the opportunity to use Internet-based instruments to assess physical activity in large populations with enhanced accuracy, minimal logistic burden, and reduced time and costs. Although a wide range of physical activity questionnaires is established, few instruments are Web-based or provide information about the type, frequency, and duration of physical activity across the entire day. Previous Web-based [7], computer-based [8-11], and cell-phone-based [12] 24-h physical activity recalls were developed in English [8-12] and Japanese [7]. Those instruments showed high validity correlation coefficients of .87 to .91 for total energy expenditure estimates when compared against doubly-labeled water [7] and multi-sensors [8], low to moderate accelerometer-based validation correlation coefficients of .36 to .72 for total energy expenditure [9,10], and of .26 to .59 for total time spent in sedentary and moderate to vigorous intensity activities [10-12].

The purpose of this study was to develop a computer-based 24-h physical activity recall (cpar24) instrument and to evaluate its concurrent validity, test-retest reliability, and usability with respect to the recalled 24-h period based on a population-based sample from a pilot study of the German National Cohort [13]. The cpar24 represents part of the physical activity assessment in the German National Cohort [13], a population-based prospective study of 200,000 men and women aged 20-69 years, which was initiated in 2014.

## Methods

### Study Protocol and Participants

The study was conducted from July to August, 2011 as part of a pilot study of the German National Cohort and included a random sample of 67 healthy participants (34 women and 33 men) aged 22 to 70 years from Regensburg, Germany. Exclusion criteria were lack of language skills, no Internet access, no computer experience, and unwillingness to wear an accelerometer. Sixty-seven participants took part in the study by completing the cpar24 twice during their visit at the study center. Of those, 49 subjects (73%, 49/67) wore the GT3X+ accelerometer for 3 days and subsequently completed the cpar24 a third time at home. Fifty-three subjects (79%, 53/67) responded to the usability survey. The study protocol was approved by the ethics committee of Regensburg University, and all participants provided written informed consent.

### Description of the Computer-Based 24-hour Physical Activity Recall (cpar24) Instrument

The cpar24 is a self-administered, computer-based, Web-based-accessible 24-h physical activity recall instrument designed to assess detailed information about the specific types, durations, and intensities of active and sedentary behaviors on the previous day (midnight to midnight). It was developed to be easy to administer, with minimal user training, and a completion time of 30 min or less for the majority of participants. Specifically, the cpar24 guides a participant to select, in chronological order, specific activities performed throughout the previous 24-h period from a list of 262 activities that are divided into the 13 following broad categories: (1) sleeping and reclining; (2) personal care; (3) food preparation and eating; (4) walking, transportation, and traveling; (5) household chores; (6) occupational activity; (7) shopping, errands, and appointments; (8) leisure and hobbies; (9) sports; (10) family life and social activities; (11) outdoor activities; (12) lawn and garden; and (13) miscellaneous activities. In addition, the respondent may refer to an alphabetical list of activities using a search function or select a specific activity using a search box. The response categories and follow-up probes were designed to allow the respondent to select broad activity classifications (eg, sports) followed by questions regarding more specific aspects of the activity within the category reported (eg, soccer). The participant can view his or her responses through an interactive calendar that allows response editing by dragging or dropping response items.

Once an activity is selected, the respondent is asked to indicate the start and end times of the activity in durations of 5 min or more. A minimal bout length of 5 min was chosen to facilitate reporting of activities of short duration. The respondent is able to enter 2 activities during the same 5 min time period, in line with the recommendation that physical activity diaries should include main activities as well as activities performed in parallel [14]. For activities that require a ranking of intensity (eg, cycling and Nordic walking), the respondent is asked to indicate the level of effort using categories of light, medium, and hard intensities. For activities that can be performed either standing or sitting or a combination of standing and sitting, the respondent is requested to specify the ratio of standing to sitting time using a scale from 0% to 100%. Each activity reported is assigned a metabolic equivalent of task (MET) value based on the most recent compendium of physical activities published by Ainsworth et al [15].

Respondents are asked to fully complete the recall before ending the session. To ensure complete data entry, a review of all items reported is provided, and the respondent is informed about missing or incomplete activity entries (ie, time gaps) with the option of adding new activity items in order to arrive at the desired total amount of 1440 min (=24 h) of logged activities per day. At the end of the recall, a brief survey on respondent burden and usability is administered. Specifically, the respondent is asked to report the time needed to complete the recall and to respond to the following 6 questions, with response options ranging from 1 (excellent) to 6 (unsatisfactory): (1) “How well were you able to recall activities performed yesterday?” (2) “How helpful was the user’s manual?” (3) “How helpful were the broad activity categories (eg, household chores, outdoor activities) to find a specific activity?” (4) “How would you rate the overall ease of using the cpar24?” (5) “How well were you able to navigate the cpar24 interface?” and (6) “Do you like the design of the cpar24?”

### Criterion Measure of Physical Activity

Accelerometry is an established simple, noninvasive, and cost-efficient method for assessing physical activity in a detailed and objective manner [16,17] and was therefore selected as criterion measure. We used the GT3X+ accelerometer (ActiGraph, LLC, Pensacola, FL, USA). This device measures motion in 3 axes with a sampling rate of 100 Hz and the output is expressed as counts per epoch. Participants wore the GT3X+ accelerometer over a 3-day period and subsequently completed the cpar24 at home on the third day, recalling their previous day's activity, that is, their activities on the second day of accelerometer measurement. Accelerometers were fitted by skilled personnel at the study center and worn on a belt at the natural waistline on the right hip in line with the right axilla. Participants were instructed to wear the monitor at all times (day and night) except during swimming, sauna, and martial arts and to report the number and reasons of wear interruptions in a specific document. Accelerometer data were downloaded using the ActiLife v5.6.4 Firmware v2.1.0 software (ActiGraph, LLC, Pensacola, FL, USA) and were subsequently checked to ensure that the device had been functioning properly. Accelerometer data with less than 12 h (50%) of wear time were excluded from analysis. Since the second day of accelerometer monitoring covered the cpar24 recall period, only data referring to that 24-h time period were included.

### Statistical Methods

To examine the validity of the cpar24 in relation to accelerometry, we compared cpar24 data with accelerometer data among participants with complete data from both assessment methods. For cpar24 data, activity intensities were classified as sedentary (<1.5 MET), light (1.5-2.9 MET), and moderate to vigorous (≥3.0 MET). For accelerometer data, the activity intensity classification was based on the Freedson formula [18] in combination with the 100 counts per minute cut-off for sedentary activities as suggested by Matthews et al [19], classifying the intensity of activities as sedentary (less than 100 counts per min), light (100 to 1951 counts per min), and moderate to vigorous (1952 or more counts per min). In subanalyses, we assessed the validity of the cpar24 stratified by age (<60 years, ≥60 years), gender (men, women), and body mass index (BMI: <25 kg/m^2^, ≥25 kg/m^2^).

We assessed the reliability of the cpar24 instrument based on two cpar24 recalls from the same 24-h period, the completions of which were separated by approximately 3 h. We used the first cpar24 recall as criterion measure to assess the reliability of the instrument in the entire sample and in subgroups defined by age, gender, and body mass index (BMI). To assess the usability of the cpar24, we evaluated the 6-item usability questionnaire stratified by age, gender, and BMI.

All statistical analyses were conducted using nonparametric methods, including Spearman correlations, median, and rank comparisons. In particular, we tested if the median total time spent in sedentary, light or moderate to vigorous activities varied according to the assessment method (accelerometer vs cpar24) using the Wilcoxon signed rank test. In addition, we computed the difference in the total time spent in a specific physical activity intensity level between the two assessments (accelerometry vs cpar24) for each participant, and we tested if that difference varied across strata defined by the participants’ age, gender, and BMI using the Wilcoxon rank sum test. We also generated Bland-Altman plots [20] to examine the agreement between the activity variables. We conducted 2-sided statistical tests at a significance level of 5%. All analyses were performed using the statistical software R, version 3.2.3 [21].

## Results

### Participants’ Characteristics

The study sample showed a nearly equal gender distribution (34 women and 33 men, Table 1). The mean age of the participants was 52 years (range=22-70 years), and their mean BMI was 26.1 kg/m^2^ (range=18.1-41.2 kg/m^2^).

### Validity of the Computer-Based 24-Hour Physical Activity Recall (cpar24) Instrument Estimates

The cpar24 and accelerometer estimates of the total activity duration were modestly positively correlated, showing Spearman correlations of .54 for sedentary activity, .46 for light activity, and .50 for moderate to vigorous activity (Table 2). However, the cpar24 underestimated the time spent in light activities by −123 min (corresponding to −28%, *P* difference <.001), and it overestimated moderate to vigorous activity by 89 min (corresponding to +353%, *P* difference <.001) when compared with accelerometer measurements. In contrast, the 2 assessment methods agreed with respect to time spent sedentary (*P* difference=.39). The pattern of agreement of total time spent in sedentary, light, and moderate to vigorous activities was not affected by age, gender, and BMI of participants (all *P* difference ≥.23).

**Table 1:** Characteristics of the participants included in the reliability, validity, and usability studies of the computer-assisted 24-hour physical activity recall (cpar24) instrument.

Variable		Reliability study	Validity study	Usability study
**Participants, n (%)**				
	Total	67 (100)	49 (100)	53 (100)
	Men	33 (49)	24 (49)	26 (49)
	Women	34 (51)	25 (51)	27 (51)
**Age, years**				
	Minimum	22	22	22
	Maximum	70	69	69
	Mean	52	50	53
	Standard deviation	13	13	13
**Body mass index, kg/m^2^**				
	Minimum	18.1	18.2	18.2
	Maximum	41.2	41.2	41.2
	Mean	26.1	26.1	26
	Standard deviation	4.4	4.7	4.1

**Figure 1:**
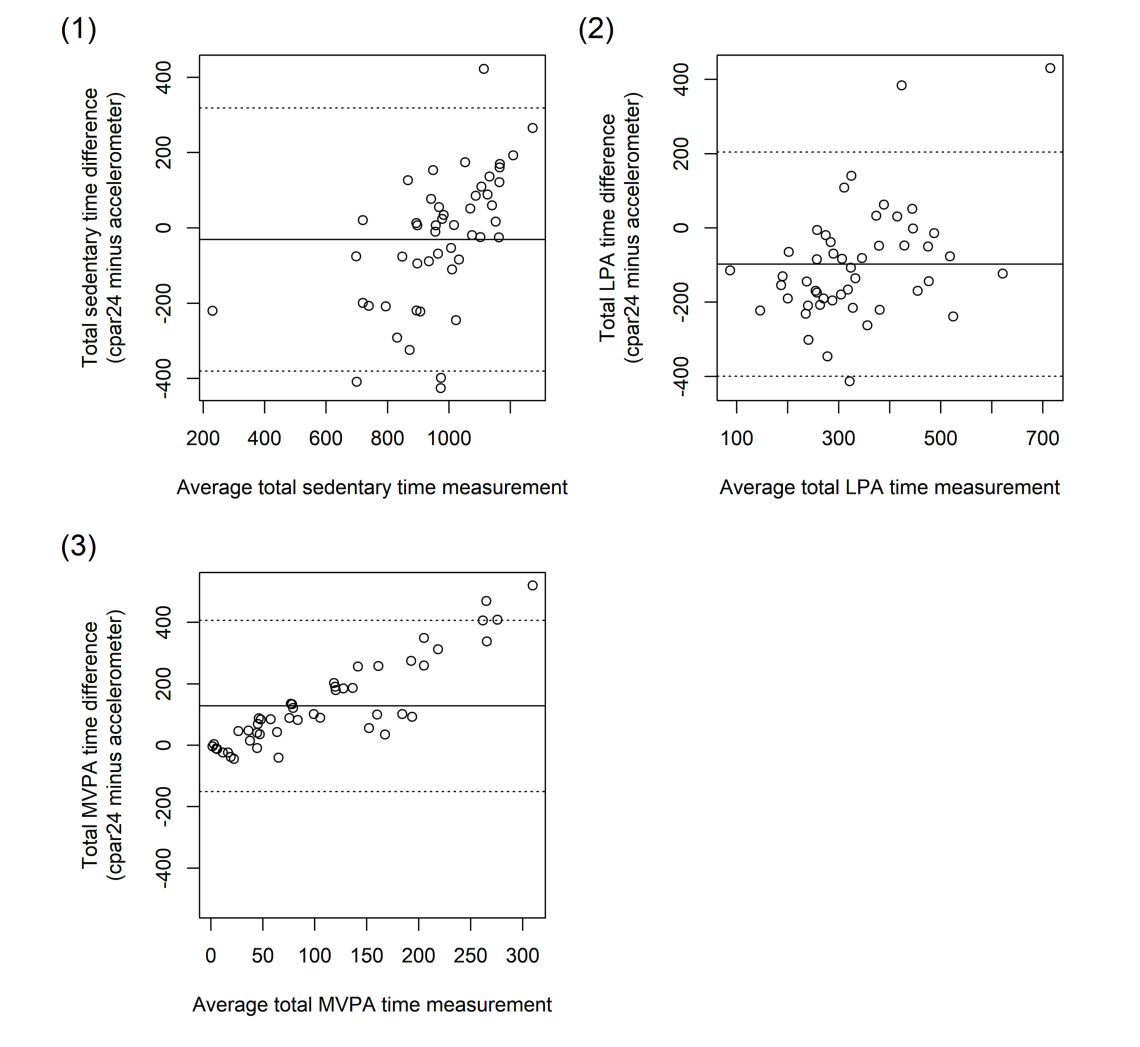
Bland Altman plots comparing computer-based 24-hour physical activity recall (cpar24) instrument data against accelerometry data of the 49 participants of the validity study with respect to (1) the total time spent in sedentary activities, (2) the total time spent in light physical activities, and (3) the total time spent in moderate to vigorous physical activities. LPA=light physical activity; MVPA=moderate to vigorous physical activity.

Bland-Altman plots illustrated the previously described bias regarding the assessments of light activity and moderate to vigorous activity (Figure 1). The difference between the estimates increased with the magnitude of the estimates. This also held true for sedentary behavior (Figure 1) despite the previously observed comparability of the corresponding median values (Table 2). According to the Bland-Altman analyses, the mean bias and limits of agreement (LoA) were −31 min (LoA=−380 to +319 min) for sedentary time, −98 min (LoA=−399 to +204 min) for light intensity physical activity, and +128 min (LoA=−151 to +407 min) for moderate to vigorous intensity physical activity.

### Reliability of the cpar24

Reliability analyses (Table 3) yielded moderate to strong Spearman correlations for time spent sedentary (*r*=.75), light (*r*=.65), and moderate to vigorous activities (*r*=.92). In the reliability analyses, no systematic bias was observed between the two cpar24 assessments of the total durations of sedentary, light, and moderate to vigorous activities (all *P* difference ≥.60). In general, age, gender, and BMI of participants did not influence the results (all *P* difference ≥.09). However, for moderate to vigorous physical activity, the median difference between the two assessments varied statistically significantly across age groups even though the absolute difference was not substantial. Specifically, the median difference between the two 24-h physical activity recalls with respect to total duration of moderate to vigorous physical activities was null among people aged less than 60 years, and it was 8 min among people aged 60 years or more; *P* difference=.04). Similarly, the average MET values were comparable across the two 24-h recalls, yielding median values of 1.71 and 1.69 for the first and second 24-h recall, respectively (*P* difference=.34 as assessed by the Wilcoxon signed rank test; Spearman correlation=.91).

**Table 2:** Comparison of total time spent in sedentary, light, and moderate to vigorous activity during the 24-h period as assessed by accelerometery and by computer-based 24-h physical activity recall (cpar24) instrument.

Stratum and variable		Sedentary activity^a^	Light activity^a^	Moderate to vigorous activity^a^
Total time during 24-h period				
**All participants**				
	Median total time based on accelerometer data, in min	1004	377	30
	Median total time based on cpar24^e^ data, in min	980	265	145
	Median difference between cpar24 and accelerometer total time^b^, in min (and in %)	7 (+1)	−123 (−28)	89 (+353)	
*P* difference^c^		.39	<.001	<.001
Spearman correlation		.54	.46	.50
**Participants aged < 60 years**				
	Median total time based on accelerometer data, in min	978	391	30
	Median total time based on cpar24 data, in min	980	265	120
	Median difference between cpar24 and accelerometer total time^b^, in min (and in %)	7 (+1)	−130 (−31)	85 (+353)
*P* difference^c^		.66	<.001	<.001
Spearman correlation		.56	.48	.46
**Participants aged ≥ 60 years**				
	Median total time based on accelerometer data, in min	1022	361	42
	Median total time based on cpar24 data, in min	968	255	150
	Median difference between cpar24 and accelerometer total time^b^, in min (and in %)	−36 (−3)	−102 (−21)	96 (+391)
*P* difference^c^		.26	.03	.003
Spearman correlation		.37	.33	.73
*P* value for the influence of age on the difference between cpar24 and accelerometer data^d^		.46	.55	.38
**Men**				
	Median total time based on accelerometer data, in min	1014	361	39
	Median total time based on cpar24 data, in min	985	182	148
	Median difference between cpar24 and accelerometer total time^b^, in min (and in %)	8 (+1)	−149 (−49)	92 (+350)
*P* difference^c^		.82	<.001	<.001
Spearman correlation		.65	.47	.62
**Women**				
	Median total time based on accelerometer data, in min	978	400	30
	Median total time based on cpar24 data, in min	930	305	125
	Median difference between cpar24 and accelerometer total time^b^, in min (and in %)	−24 (−2)	−83 (−21)	85 (+400)
*P* difference^c^		.20	.01	<.001
Spearman correlation		.50	.40	.40
*P* value for the influence of gender on the difference between cpar24 and accelerometer data^d^		.27	.26	.99
**Participants with a BMI^f^<25.0 kg/m^2^**				
	Median total time based on accelerometer data, in min	972	381	38
	Median total time based on cpar24 data, in min	992	265	110
	Median difference between cpar24 and accelerometer total time^b^, in min (and in %)	17 (+2)	−118 (−28)	70 (+192)
*P* difference^c^		.84	.005	<.001
Spearman correlation		.56	.54	.59
**Participants with a BMI≥25.0 kg/m^2^**				
	Median total time based on accelerometer data, in min	1017	367	30
	Median total time based on cpar24 data, in min	950	255	145
	Median difference between cpar24 and accelerometer total time^b^, in min (and in %)	−53 (−5)	−135 (−31)	122 (+600)
*P* difference^c^		.23	.001	<.001
Spearman correlation		.48	.24	.49
*P* value for the influence of the BMI on the difference between cpar24 and accelerometer data^d^		.36	.95	.23

^a^For accelerometer counts, we classified the physical activity intensity according to the Freedson formula combined with the 100 counts per min cut-off suggested by Matthews: sedentary activity (counts per min<100), light physical activity (100≤counts per min<1952), moderate to vigorous physical activity (1952≤counts per min); for self-reported physical activity (cpar24), we classified the physical activity intensity according to the corresponding metabolic equivalent of task (MET) value from the Ainsworth compendium: sedentary activity (MET≤1.5), light physical activity (1.5<MET<3.0), moderate to vigorous physical activity (3.0≤MET).

^b^Please note that the median of the difference between 2 variables does not necessarily correspond to the difference between the medians of the 2 variables.

^c^We tested if the median total time spent in sedentary, light or moderate to vigorous activities varied according to the assessment method (accelerometer vs cpar24) using the Wilcoxon signed rank test.

^d^We computed the difference in the total time spent in a specific physical activity intensity level between the two assessments (accelerometry vs cpar24) for each participant, and we tested if that difference varied across the two strata of participants using the Wilcoxon rank sum test.

^e^cpar24: computer-based 24-h physical activity recall.

^f^BMI: body mass index.

**Figure 2:**
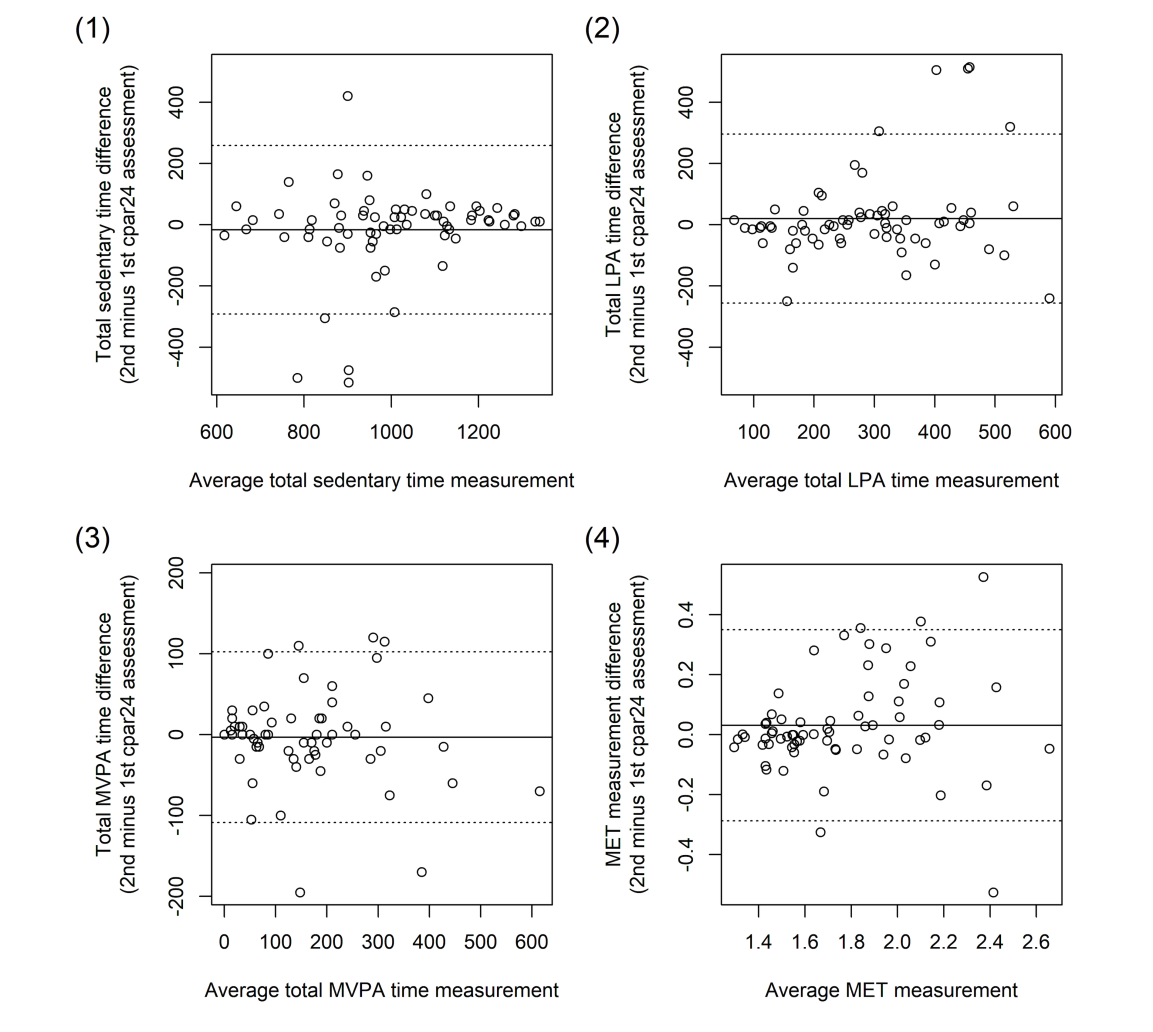
Bland Altman plots comparing data from the second computer-based 24-hour physical activity recall (cpar24) against data from the first cpar24 recall among the 67 participants of the reliability study with respect to (1) the total time spent in sedentary activities, (2) the total time spent in light physical activities, (3) the total time spent in moderate to vigorous physical activities, and (4) the average metabolic equivalent of task (MET) value. LPA=light physical activity; MVPA=moderate to vigorous physical activity; MET=metabolic equivalent of task.

In agreement with the reliability analyses from Table 3, Bland-Altman plots did not indicate any systematic bias for total time spent in sedentary, light, and moderate to vigorous activities and for the average MET value for the entire 24-h period (Figure 2). According to the Bland-Altman analyses, the mean bias and limits of agreement were −17 min (LoA=−292 to +259 min) for sedentary time, +20 min (LoA=−256 to +296 min) for light intensity physical activity, −3 min (LoA=−109 to +102 min) for moderate to vigorous intensity physical activity, and 0.0 METs (LoA=−0.3 to +0.3 METs) for the average MET value.

### Usability of the cpar24

**Figure 3:**
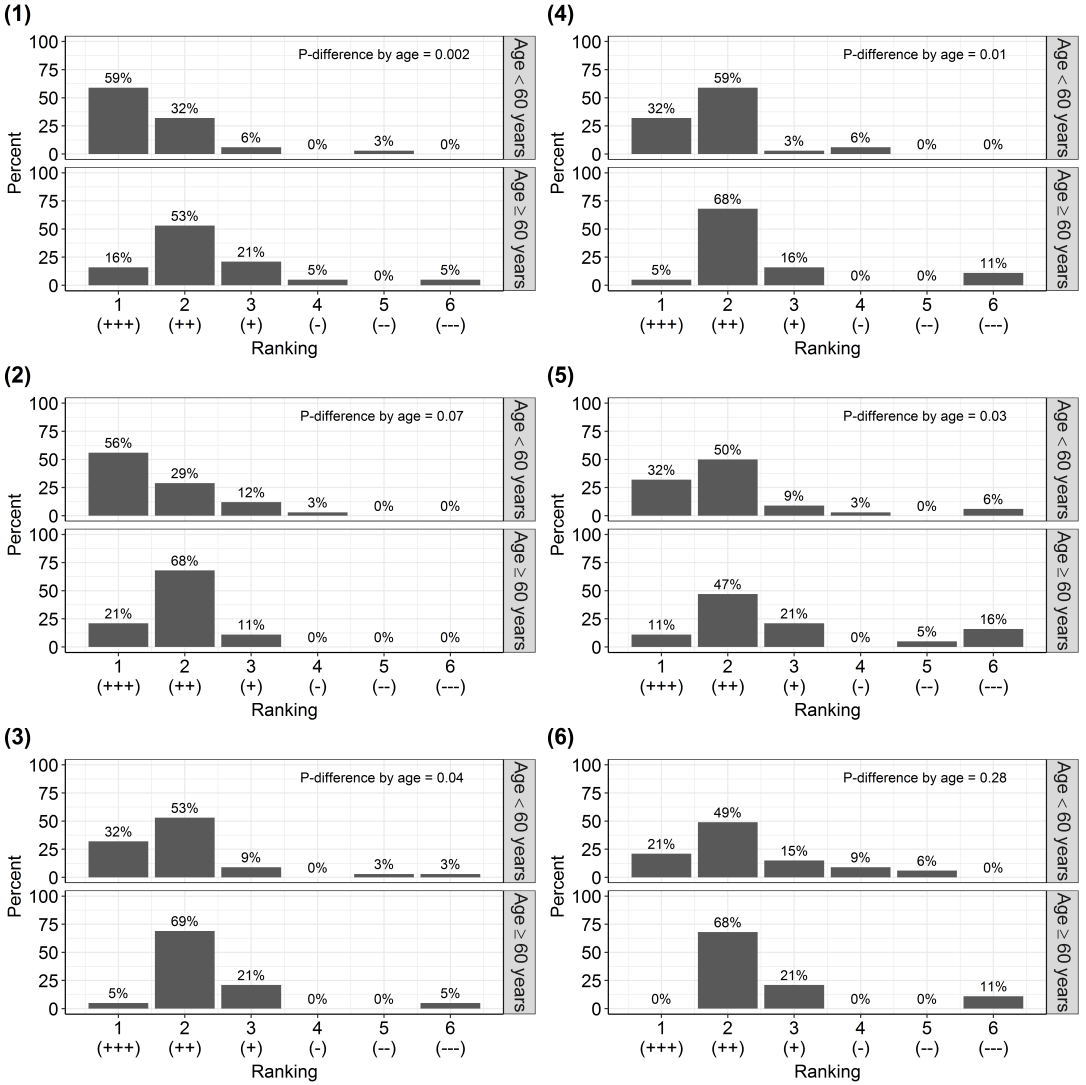
Proportion of the 53 participants of the usability study awarding the ranks 1 (excellent) to 6 (unsatisfactory) to the six items: (1) “How well were you able to recall activities performed yesterday?”, (2) “How helpful was the user’s manual?”, (3) “How helpful were the broad activity categories (eg, household chores, outdoor activities) to find a specific activity?”, (4) “How would you rate the overall ease of using the cpar24?”, (5) “How well were you able to navigate the cpar24 interface?”, and (6) “Do you like the design of the cpar24?”, stratified by age group. The heterogeneity across age was assessed using the Wilcoxon rank sum test. Please note that the result of the Wilcoxon rank sum test was not similar for items (3) and (6) in spite of comparable patterns between the age-specific distributions of rankings for items (3) and (6). The reason was that the Wilcoxon rank sum test assessed the difference between medians and not between distributions. If the difference in distributions across age groups had been tested using Fisher exact test, statistically significant difference in the distributions of rankings by age group would have been observed for all items except for item (5).

The usability of the cpar24 varied according to age. When considering participants aged less than 60 years, 82% to 91% rated the usability of the cpar24 as “excellent” or “good” with regards to their ability to recall activities performed during the previous 24 h, to find specific activities within broad activity categories, to rate the overall ease of using the cpar24, and to navigate the cpar24 interface. By comparison, when considering participants aged 60 years or more, only 58% to 74% rated the cpar24 as “excellent” or “good” (*P* difference by age <.05 for all of the aforementioned usability ratings, Figure 3). In contrast, no statistically significant difference was observed between the ratings of participants aged less than 60 years and the rating of participants aged 60 years or more with respect to the usefulness of the user’s manual and the appeal of the cpar24 design, which received “excellent” or “good” ratings from 70% to 85% of participants aged less than 60 years and 68% to 89% of participants aged 60 years or more. In contrast, gender (all *P* difference ≥.07) and BMI (*P* difference ≥ .08) did not affect the ratings for any of the usability survey items after stratification by age. Participants completed the cpar24 within an average of 25 min (median, range=10-53 min, interquartile range=20-30 min).

**Table 3:** Comparison of total time spent in sedentary, light, and moderate to vigorous activity during the 24-hour period across the two 24-hour physical activity recalls (cpar24).

Stratum and variable		Sedentary activity^a^	Light activity^a^	Moderate to vigorous activity^a^
Total time during 24-h period				
**All participants**				
	Median total time based on 1st 24-h recall, in min	1010	265	120
	Median total time based on 2nd 24-h recall, in min	990	300	115
	Median difference between 1st and 2nd 24-h recall^b^, in min (and in %)	10 (+1)	−5 (−1)	0 (+0)
*P* difference^c^		.60	.89	.68
Spearman correlation		.75	.65	.92
**Participants aged < 60 years**				
	Median total time based on 1st 24-h recall, in min	1035	250	105
	Median total time based on 2nd 24-h recall, in min	1025	295	100
	Median difference between 1st and 2nd 24-h recall^b^, in min (and in %)	10 (+1%)	0 (+0%)	0 (+0%)
*P* difference^c^		.88	.55	.10
Spearman correlation		.75	.58	.93
**Participants aged ≥60 years**				
	Median total time based on 1st 24-h recall, in min	1002	288	120
	Median total time based on 2nd 24-h recall, in min	990	305	130
	Median difference between 1st and 2nd 24-h recall^b^, in min (and in %)	25 (+3%)	−18 (−9%)	8 (+0%)
*P* difference^c^		.55	.24	.22
Spearman correlation		.76	.87	.83
*P* value for the influence of age on the difference between cpar24 and accelerometer data^d^		.45	.21	.04
**Men**				
	Median total time based on 1st 24-h recall, in min	1010	240	160
	Median total time based on 2nd 24-h recall, in min	1025	255	120
	Median difference between 1st and 2nd 24-h recall^b^, in min (and in %)	25	−5	0
*P* difference^c^		.15	.61	.23
Spearman correlation		.75	.64	.94
**Women**				
	Median total time based on 1st 24-h recall, in min	1012	282	85
	Median total time based on 2nd 24-h recall, in min	970	320	82
	Median difference between 1st and 2nd 24-h recall^b^, in min (and in %)	−10 (−1)	0 (+0)	0 (+0)
*P* difference^c^		.49	.80	.37
Spearman correlation		.75	.66	.92
*P* value for the influence of gender on the difference between 1st and 2nd 24-h recall^d^		.17	.57	.09
**Participants with a BMI<25.0 kg/m^2^**				
	Median total time based on 1st 24-h recall, in min	1035	232	128
	Median total time based on 2nd 24-h recall, in min	990	298	128
	Median difference between 1st and 2nd 24-h recall^b^, in min (and in percent)	5	0	0
*P* difference^c^		.73	.32	.20
Spearman correlation		.82	.65	.96
**Participants with a BMI≥25.0 kg/m^2^**				
	Median total time based on 1st 24-h recall, in min	1000	290	105
	Median total time based on 2nd 24-h recall, in min	990	300	70
	Median difference between 1st and 2nd 24-h recall^b^, in min (and in %)	15 (+1)	−15 (−7)	0 (+0)
*P* difference^c^		.29	.25	.73
Spearman correlation		.69	.71	.89
*P* value for the influence of the BMI on the difference between 1st and 2nd 24-h recall^d^		.18	.10	.21

^a^For self-reported physical activity (cpar24), we classified the physical activity intensity according to the corresponding metabolic equivalent of the MET value from the Ainsworth compendium: sedentary activity (MET≤1.5), light physical activity (1.5<MET<3.0), moderate to vigorous physical activity (3.0≤MET).

^b^Please note that the median of the difference between 2 variables does not necessarily correspond to the difference between the medians of the 2 variables.

^c^We tested if the median total time spent in sedentary, light or moderate to vigorous activities varied between the 1st and 2nd 24-h recall using the Wilcoxon signed rank test.

^d^We computed the difference in the total time spent in a specific physical activity intensity level between the 1st and 2nd 24-h recall for each participant, and we tested if that difference varied across the two strata of participants using the Wilcoxon rank sum test.

^e^cpar24: computer-based 24-h physical activity recall.

## Discussion

### Principal Findings

We assessed the validity, reliability, and usability of the cpar24. Information from the cpar24 was modestly positively correlated with information from accelerometry regarding estimates of the total time spent in sedentary, light, and moderate to vigorous activities. However, as compared with accelerometry, the cpar24 tended to overestimate time spent in moderate to vigorous activities while underestimating time spent in light activities. In contrast, we observed strong positive correlations and no systematic bias between repeated cpar24 assessments. Participants assigned high rankings to the usability of the cpar24, particularly those younger than age 60 years.

### Relevance of Short-Term Physical Activity Recalls to Assess Physical Activity in Epidemiologic Studies

Most available physical activity questionnaires assess the intensity, frequency, and duration of common physical activities performed during the past week, past month, or past year [22]. In the past 20 years, assessments of physical activities of the previous week have become the most prevalent form [23], most likely because estimates of recent past activity patterns (past 24 h, past 7 days) are more accurate than estimates of average physical activity levels representative of longer time periods (eg, past month, past year) [4], leading to an average accelerometer based validity correlation coefficient of .41 for previous week questionnaires as compared with an average correlation coefficient of .30 for previous year assessments [22].

To improve the accuracy of estimates, there have been recommendations to administer multiple short-term physical activity recalls (past 24 h, past 7 days) and to average activity levels over those recalls when using self-reports in large-scale epidemiologic studies [4]. In addition, thanks to recent technologic advances, accelerometers can now be employed to measure short-term physical activity (24 h to 7 days) in large studies [24]. The objective nature of accelerometer measurements represents a potential advantage over self-reported physical activity because the latter may be prone to recall bias and to measurement error resulting from the difficulty of classifying physical activity intensity and from reporting socially desirable physical activity patterns. However, as compared with questionnaires, accelerometers come at the costs of greater logistic burden, increased data complexity, and lower acceptance among participants [24]. In addition, accelerometry has difficulty in recognizing resistance components of activities [25-27], such as carrying heavy objects or ascending stairs. Furthermore, in accelerometry, low and high pass filters are used in an attempt to distinguish human acceleration from noise and from motorized acceleration, implying that accelerometers cannot detect very fast human motion [28]. For example, the ActiGraph digital filter rejects frequencies below 0.25 Hz because those frequencies are mainly associated with gravity acceleration [29] and it rejects frequencies above 2.5 Hz because those frequencies are mainly associated with motorized acceleration (eg, when traveling by car or train). The remaining frequency range of 0.25-2.5 Hz is thought to reflect human body acceleration but it can only identify gait speeds up to 12 km per h [28]. For higher gait speeds, there is an inverse relation between the true gait speed and the gait speed derived when only considering frequencies in the range of 0.25-2.5 Hz, leading to a circumstance in which frequencies from running at 16, 18, and 20 km per h resemble gait speeds of 10, 8, and 6 km per h, respectively [30,31]. However, few people achieve high gait speeds. In addition, any potential misclassification of the exact speed will not affect estimates for time spent in moderate to vigorous physical activity because even a gait speed of 6 km per h is classified as moderate to vigorous physical activity.

### Validity of Previous Short-Term Physical Activity Recalls as Compared With the Gold Standard of Doubly-Labeled Water

Studies using doubly-labeled water measurements as the gold standard to validate energy expenditure estimates obtained from short-term physical activity recalls (24 h to 7 days) and from accelerometry revealed similar validation correlation coefficients for both methods although, within each method, the validation correlation coefficients of total energy expenditure tended to be greater than the validation correlation coefficients of physical activity energy expenditure (total energy expenditure estimates from short-term physical activity recalls [7,32-39]: average correlation=.57, range=.32-.88; total energy expenditure from accelerometry [40]: average correlation=.52, range=.18-.83; physical activity energy expenditure from short-term physical activity recalls [7,38,39,41,42]: average correlation=.21, range=−.07-.68; physical activity energy expenditure from accelerometry [40]: average correlation=.39, range=−.30-.83). Similarly, there appeared to be less variation across accuracy estimates of total energy expenditure than physical activity energy expenditure for both methods when using doubly-labeled water as the gold standard (total energy expenditure from short-term physical activity recalls [7,32-35,37-39,42-48]: mean percent difference=7%, range=−27%-37%; total energy expenditure from accelerometry [40]: mean percent difference=−12%, range=−22%-1%; physical activity energy expenditure from short-term physical activity recalls [7,38,39,41,48,49]: mean percent difference=20%, range=−20%-113%; physical activity energy expenditure from accelerometry [40]: mean percent difference=−24%, range=−59%-40%). However, accelerometry tended to underestimate energy expenditure, whereas short-term physical activity recalls tended to overestimate energy expenditure.

### Validity of Previous Short-Term Physical Activity Recalls as Compared With Accelerometry

Studies validating physical activity recalls using accelerometry as the gold standard reported stronger average correlation coefficients between energy expenditure estimates (expressed as total energy expenditure, physical activity energy expenditure, average MET per hour, or physical activity MET per week) and accelerometer counts per minute from 24-h recalls than from 7-day recalls (24-h recalls [9,10,41,50,51]: average correlation=.48, range=.23-.82; 7-day recalls [41,51-55]: average correlation=.36, range=−.02-.90). In contrast, average correlations between self-report and accelerometer-based estimates for time spent in sedentary and light activities were greater for 7-day recalls than for 24-h recalls, whereas those for time spent in moderate to vigorous activities agreed across 24-h recalls and 7-day recalls (time spent in sedentary activities among 24-h recalls [10,12]: average correlation=.19, range=−.05-.59; time spent in sedentary activities among 7-day recalls [52,54-57]: average correlation=.37, range=.20-.65; time spent in light activities among 24-h recalls [11,12,58]: average correlation=.18, range=−.16-.45; time spent in light activities among a single 7-day recall [58]: correlation=.37; time spent in moderate to vigorous activities among 24-h recalls [10,11,59,60]: average correlation=.19, range=.05-.26; time spent in moderate to vigorous activities among 7-day recalls [39,55-57,60,61]: average correlation=.26, range=.06-.51). Studies comparing short-term physical activity recalls (24 h to 7 days) with accelerometry tended to report greater estimates of total energy expenditure, light activities, and moderate to vigorous activities (percent difference for total energy expenditure [50,51]: mean=+19%, range=+12%-+31%; for physical activity energy expenditure [49,53]: mean=+87%, range=+80%-+95%; for light activities [11,12,58]: mean=+36%, range=−8%-+107%; for moderate to vigorous activities [11,55,56,59-61]: mean=+260%, range=+29%-+778%). In contrast, there were as many studies overestimating sedentary activities as there were studies underestimating sedentary activities (percent difference for sedentary activities among studies overestimating sedentary activities [11,12,54]: mean=+17%, range=+11%-+27%; among studies underestimating sedentary activities [55-57]: mean=−32%, range=−44% to −13%; among all studies estimating sedentary activities [11,12,54-57]: mean=−4%, range=−44%-+27%).

### Reliability of Previous Short-Term Physical Activity Recalls

The reliability correlation coefficients of short term physical activity recalls (24 h to 7 days) appear to decrease with increasing time between replicate measurements. Replicate measurements of a specific 24-h physical activity recall separated by a time lag of 4 hours yielded a positive correlation of .99 [9,10], whereas correlation coefficients for another 24-h physical activity recall varied between .55 and .63 for a time lag of 6 months [12]. Similarly, the smaller the time lag between replicate measurements, the greater the reliability coefficient for 7-day physical activity recalls. Specifically, the reliability coefficient for a time lag of less than a week is .79 (range=.45-.99), for a time lag of 1-4 weeks it is .63 (range=.22-.91), and for a time lag of 2-12 months it is .50 (range=.33-.65) [23]. In contrast, reliability coefficients of 12-month physical activity recalls appear to be less sensitive to the length of the period between measurements. The reliability coefficient for a time lag of less than 1 month is .68 (range=.17-.99) and for a time lag of 2-12 months it is .72 (range=.65-.78) [23]. Reliability coefficients of 7-day physical activity recalls administered less than 1 week apart appeared to be greater for sedentary (mean reliability coefficient=.81, range=.71-.91) [23] than for moderate to vigorous physical activity (mean reliability coefficient=.76, range=.45-.99) [23] and for total energy expenditure (mean reliability coefficient=.73, range=.54-.93) [23]. Two previous studies [9,10] investigated the reliability of a single 24-h physical activity recall with measurements taken 4 hours apart and reported reliability coefficients of .99 each for time spent in moderate to vigorous physical activity and for total energy expenditure. Those studies [9,10] did not report reliability coefficients for total sedentary activity but provided data for sleep (*r*=.99), screen time (*r*=.99), and the complement of sedentary time (nonsedentary time, *r*=.99).

### Reliability of the cpar24 in Comparison With Previous Short-Term Physical Activity Recalls

In our study, the reliability correlation coefficients for the total time spent in sedentary (*r*=.75), light (*r*=.65), and moderate to vigorous (*r*=.92) activities, and the reliability correlation coefficient for total energy expenditure (*r*=.91, assessed as average MET per h) were in the top range of reliability coefficients observed previously for 7-day physical activity recalls administered less than 1 week apart (average correlation=.76, range=.45-.99) [23]. However, the reliability correlation coefficients for our 24-h physical activity recall ranging between .65 and .92 were smaller than those reported for a previous 24-h physical activity recall (all *r*=.99) [9,10]. To our knowledge, reliability correlation coefficients for additional previous 24-h physical activity recalls are currently not available for further comparison. In line with a previous 24-h recall [9], no statistically significant differences emerged between estimates of average MET and time spent in specific activity intensities obtained from two 24-h physical activity recalls, the second of which was completed 3 hours after completion of the first recall.

### Validity of the cpar24 in Comparison With Previous Short-Term Physical Activity Recalls

In our validation study, we deliberately refrained from comparing MET values derived from accelerometer counts with MET values derived from the 24-h physical activity recall because neither method provides accurate MET estimates. In particular, the derivation of MET values from accelerometer counts is challenging, and no conversion rule has been proven universally valid, not even with respect to treadmill walking or running, the discipline for which most formulae were derived [18,25-27,62-69]. Similarly, divergences of measured MET values from the Ainsworth MET values in either direction have been reported for a wide range of activities, including walking or running, ascending or descending stairs, and moving heavy objects [15,70], suggesting that representing a specific activity by a single MET value is challenging.

We found that the validity correlation coefficients for our 24-h physical activity recall for the total time spent in sedentary (*r*=.54), light (*r*=.46), and moderate to vigorous activity (*r*=.50) were superior to the average validity correlation coefficients reported for previous 24-h physical activity recalls evaluated against accelerometry (validity correlation coefficient for sedentary activity [10,12]: mean=.19, range=−.05-.59; for light physical activity [11,12,58]: mean=.18, range=−.16-.45; for moderate to vigorous physical activity [10,11,59,60]: mean=.19, range=.05-.26). The validity correlation coefficients of our 24-h physical activity recall were also in the top range when compared with previous 7-day physical activity recalls evaluated against accelerometry (validity correlation coefficient for sedentary activity [52,54-57]: mean=.37, range=.20-.65; for light physical activity [58]: mean=.37; for moderate to vigorous physical activity [39,55-57,60,61]: mean=.26, range=.06-.51).

When comparing cpar24 data with accelerometer data, we found that the cpar24 statistically significantly overestimated moderate to vigorous physical activity time by +353%, which was greater than the average overestimation of +260% (range=+29%-+778%) reported in 6 previous studies [11,55,56,59-61] evaluating short-term recalls (24 h to 7 days) against accelerometry. In our study, the statistically significant overestimation of moderate to vigorous physical activity corresponded to an absolute difference of 89 min, and it was compensated by a statistically significant underestimation of time spent in light activities by −123 minutes (−28%). By comparison, previous short-term physical activity recalls (24 h to 7 days) [11,12,58] tended to overestimate light activities by an average of 36% (range −8%-107%) as compared with accelerometry. In contrast to previous statistically significant over-reporting of sedentary time [11,12,54] (by an average of +17%, range=+11%-+27%) and in contrast to previous statistically significant under-reporting of sedentary time [55-57] (by an average of −32%, range=−44% to −13%), we observed a small, statistically nonsignificant overall difference of +1% between cpar24 and accelerometer estimates of sedentary time in our study. Yet, Bland Altman plots for our study revealed that the overall difference of +1% between cpar24 and accelerometer estimates of sedentary time resulted from an averaging out of over-reporting of sedentary time among sedentary participants and under-reporting of sedentary time among physically active participants. Similar observations were made in previous studies [10,54-56]. In addition, over-reporting of moderate to vigorous physical activities was stronger among physically active than sedentary participants in our and other studies [54-56,59,61].

In stratified analyses, we observed no statistically significant differences between cpar24 and accelerometer data regarding estimates of activities of various intensities across strata defined by age (aged < 60 years, aged ≥60 years), gender (men, women), and BMI (BMI<25 kg/m^2^, BMI≥25 kg/m^2^) (all *P* difference≥.23) as did several previous studies [11,12,57,59-61]. In contrast, one previous 7-day physical activity recall evaluation study [54] found that over-reporting of sedentary activities was statistically significantly greater among men as compared to women and among participants aged 18-34 years as compared with participants aged 50 years or more, whereas under-reporting of moderate to vigorous physical activities was greater among normal weight participants than among overweight and obese participants. In contrast to that study [54], another previous 7-day physical activity recall evaluation study [55] reported less over-reporting of moderate to vigorous physical activities among participants aged 18-39 years as compared with participants aged 65 years or more, whereas no statistically significant differences were seen for moderate to vigorous activities across gender and for sedentary activities across age and gender.

### Strengths and Limitations

An important strength of our study is the use of accelerometry as objective comparison criterion, which enabled us to validate our estimates of total time spent in sedentary, light, and moderate to vigorous activities. Furthermore, the inclusion of a random sample of men and women aged 22 to 70 years from the general population allowed us to demonstrate the applicability of our 24-h physical activity recall to the general population. In addition, we conducted extensive comparisons between the validity and reliability correlation coefficients observed for our 24-h recall with those reported for a wide range of existing 24-h to 7-day physical activity recalls. We found that the validity and reliability correlation coefficients of our 24-h physical activity recall were in the top range of those reported for previous 24-h to 7-day physical activity recalls.

One limitation of our study is that we were not able to validate resistance-based activities (eg, stair climbing or carrying heavy loads) and vehicle-based activities (driving a car or cycling) due to the technologic limitations of our accelerometer. To close that gap, behavior recognition methods based on simultaneous monitoring of heart rate, body heat, body motion and position, limb motion and position, foot pressure, global positioning system, and barometric pressure are currently being evaluated [71]. Furthermore, we were not able to evaluate the absolute validity of total energy expenditure estimates, which should be done in future studies. In addition, we did not investigate the within-person variation in accelerometer and cpar24 measurements across different days and different seasons, and we can therefore not comment on how many days of measurements are required to obtain reliable physical activity estimates for a specific study period. Further studies are required to investigate the validity and reliability of cpar24 recalls to estimate average physical activity levels for longer study periods, to examine the influences of season and day of the week on the validity and reliability of those estimates, and to compare those estimates against estimates obtained from physical activity questionnaires covering the same study period.

### Conclusions

In conclusion, our cpar24 is a feasible, valid, reliable and user-friendly assessment of physical activity in adults. It provides estimates of total energy expenditure and time spent in sedentary, light, and moderate to vigorous activities with above-average validity correlation coefficients of .46 to .54 as compared with previous 24-h recall instruments. While we were able to establish the relative validity of our instrument as compared with accelerometer measurements, future studies are needed to verify the absolute validity of our cpar24.

## Abbreviations

BMI: body mass index
cpar24: computer-based 24-hour physical activity recall instrument
MET: metabolic equivalent of task
